# The Roles of Sex, Mass and Individual Specialisation in Partitioning Foraging-Depth Niches of a Pursuit-Diving Predator

**DOI:** 10.1371/journal.pone.0079107

**Published:** 2013-10-21

**Authors:** Norman Ratcliffe, Akinori Takahashi, Claire O’Sullivan, Stacey Adlard, Philip N. Trathan, Michael P. Harris, Sarah Wanless

**Affiliations:** 1 British Antarctic Survey, Cambridge, United Kingdom; 2 National Institute of Polar Research, Tokyo, Japan; 3 Centre for Ecology and Hydrology, Penicuik, United Kingdom; Hokkaido University, Japan

## Abstract

Intra-specific foraging niche partitioning can arise due to gender differences or individual specialisation in behaviour or prey selection. These may in turn be related to sexual size dimorphism or individual variation in body size through allometry. These variables are often inter-related and challenging to separate statistically. We present a case study in which the effects of sex, body mass and individual specialisation on the dive depths of the South Georgia shag on Bird Island, South Georgia are investigated simultaneously using a linear mixed model. The nested random effects of trip within individual explained a highly significant amount of the variance. The effects of sex and body mass were both significant independently but could not be separated statistically owing to them being strongly interrelated. Variance components analysis revealed that 45.5% of the variation occurred among individuals, 22.6% among trips and 31.8% among Dives, while R^2^ approximations showed gender explained 31.4% and body mass 55.9% of the variation among individuals. Male dive depths were more variable than those of females at the levels of individual, trip and dive. The effect of body mass on individual dive depths was only marginally significant within sexes. The percentage of individual variation in dive depths explained by mass was trivial in males (0.8%) but substantial in females (24.1%), suggesting that differences in dive depths among males was largely due to them adopting different behavioural strategies whereas in females allometry played an additional role. Niche partitioning in the study population therefore appears to be achieved through the interactive effects of individual specialisation and gender upon vertical foraging patch selection, and has the potential to interact in complex ways with other axes of the niche hypervolume such as foraging locations, timing of foraging and diet.

## Introduction

Competition for food has profound influences upon animal foraging ecology, population regulation, community structure and speciation [1-3]. Intra-specific competition occurs among conspecifics and may be reduced via partitioning along several axes of the niche hypervolume, including diet [4-6], timing of foraging [7,8] and foraging location [9-11]. Such partitioning can occur according to sex [12], ontogeny [13-15] or individual specialisation [16]. These in turn might be explained by body size in species where sexual size dimorphism occurs, growth is slow or indeterminate or there is substantial phenotypic variation in the size of individuals [17-19]. The traits associated with niche partitioning among sexes and individuals are therefore manifold, inter-related and challenging to separate, although this is now increasingly tractable with the development of appropriate statistical methodology [20,21].

Colonial, central-place foraging animals are particularly suited for studies of intra-specific niche partitioning. In such systems, large numbers of animals are obliged to engage in scramble competition for resources within a limited area defined by their maximum foraging ranges from the colony [22,23], resulting in high levels of intra-specific competition for food that provides the selective impetus for niche partitioning to arise. Individuals at a colony are also exposed to identical environmental conditions and have the opportunity to access the same shared resources. As such, any variation in diet or behaviour among individuals must arise from specialisation in foraging strategies, unlike in dispersed populations where it may arise from individuals having different exposure to spatially heterogeneous resources or conditions [21].

Blue-eyed shag is a taxonomic group comprising 13 sibling cormorant species that have a circumpolar distribution on islands between 30°S and 70°S: South Georgia shags *Phalacrocorax georgianus* are a species within the complex that are found on South Georgia, the South Sandwich Islands and the South Orkney Islands [24]. All members of the taxon are colonial seabirds with short foraging ranges that dive to prey on a variety of benthic fish and invertebrates [25]. Blue eyed shags are sexually size dimorphic, with males being larger than females and also diving to greater depths [26-29]. Two studies have also found evidence of individual variation in dive depths within sexes [30,31]. Studies have also found that their dive depths increase with body mass [31,32] so variability in mass both among and within genders has the potential to explain the sex- and individual-specific variation in dive-depths [26]. Blue-eyed shags are therefore an excellent model for studying the relative contributions of sex, individual specialisation and mass to variation in foraging behaviour. 

Studies of the influence of sex on dive depths have been conducted for five species in the blue-eyed shag complex, but not for South Georgia shags: this study fills this knowledge gap and provides valuable comparative information. No previous studies have analysed the effects of sex, mass and individual variation on the dive depths of blue-eyed shags within a single statistical framework, and most have been based on small sample sizes, making robust inference difficult to draw. In this study, we elucidate the degree to which variation in the dive depths of South Georgia shags are explained by sex, individual variation and body mass using contemporary analytical methods applied to data collected from a large sample of individuals over three widely spaced years.

## Methods

### Ethics statement

We noted no effects of study procedures upon the behaviour, breeding success or survival of birds during our study, and previous studies of blue-eyed shags on Bird Island and elsewhere found no change in time budgets or rates of mass loss in equipped birds compared to controls [31,33]. All fieldwork met the requirements of the BAS Animal Ethics Committee and was conducted under a permit issued by the Government of South Georgia and the South Sandwich Islands.

### Fieldwork

The study was conducted at the Wanderer Ridge colony on the south coast of Bird Island, South Georgia (54° 01’ S, 38 03° W’). Deployments were conducted when birds were incubating eggs or brooding chicks during December and January of the 1995/6, 2005/6 and 2009/10 breeding seasons. Data from 1995/6 were published in [34], those from two deep-diving males in 2005/6 were published in [35] and all data from 2009/10 are novel. Neither of the previously published papers addressed the subject matter of this paper, so the findings presented here are entirely novel. 

Off-duty birds standing by their nests were captured by ensnaring their necks using a crook or noose on a long pole. Birds were weighed to the nearest 10 g with a 5 kg Pesola spring balance and the wing length (maximum flattened chord) was measured with a wing rule to the nearest 1 mm. Birds were sexed according to vocalisations (males honk and females hiss) and wing length (males have longer wings than females) [36,37]. 

Captured birds were fitted with time-depth recorders (TDRs): Wildlife Computers Mk 5 in 1995/96 (50 g/ 2.6% of lightest mass of a bird equipped that year, Redmond, USA), Little Leonardo M190–D2GT in 2005/6 (20 g/1%, Tokyo, Japan) and CEFAS Technology G5 (standard-life model) in 2009/10 (2.7 g/0.001%, Lowestoft, UK). The Mk 5 loggers were attached to the central back feathers using waterproof tape and cable ties [34], while the G5 loggers were attached to darvic leg rings. The M190–D2GT loggers were attached to plastic netting that was then glued among the back feathers with cyanoacrylate glue (see 38 for details). All TDRs recorded pressure and time every second. All equipped birds were recaptured between one and six days later to recover the devices and download the data. 

### Data analysis

We extracted dive and trip data from the TDR data using the R package diveMove [39]. The depth data were manually zero offset corrected to define the sea-surface. The records were then calibrated (using a 2 m depth threshold) to identify dive events, which were then plotted to ensure that each started and ended at the sea surface. Dive duration and maximum depth of each dive (termed dive-depth hereafter) were extracted for each dive event. Foraging trips were defined as bouts of dive activity that were separated by a time interval of over 30 minutes during which no dives occurred. Examination of temperature records from the leg-mounted G5s confirmed that birds returned to land during any dive intervals of this duration or greater (temperatures change from low and stable in the water to higher and more variable on land [40]. These data are freely and publicly available from the British Antarctic Survey Polar Data Centre (polardatacentre@bas.ac.uk). 

We analysed the data using linear mixed models, which are advocated as the most powerful and flexible of the many methods for analysing individual repeatability [20,21], fitted in the R package nlme [41]. Depth was the response variable, sex was specified as a fixed factor, body mass as a fixed linear covariate and trip nested within individual as a random intercept effect. Models that included year and stage (incubation vs. chick-rearing) terms did not produce a significant reduction in variance (all P > 0.5) so data from these categories were pooled. The residuals of the global model were heterogeneous between the sexes (see results) so a sex-specific variance structure was applied to all models [42]. We used backward-stepwise analysis of variance for model selection, first identifying the most appropriate random effects structure using restricted maximum likelihood (REML) and then the fixed effect structure using maximum likelihood. The selected model was then refitted with REML prior to extraction of the model parameter estimates [42], which are presented ± 1 SE. 

We used variance components analysis to calculate the variance, standard deviation and proportions of total variance occurring at the levels of individual, trip within individual and dive within trip using the R package ape [43]. The proportion of variance explained by the individual variance component gives an estimate of individual repeatability or specialisation [16,21]. Variance components were first extracted from the trip nested within individual random effects model. We calculated a R^2^ approximation of the proportion of individual variation explained by the fixed effects by quantifying the proportional reduction in the individual variance component resulting from addition of the fixed effect to the random effects model [44]. We confirmed that the resulting changes in variance at the trip and dive levels were trivial, as otherwise the approximations are unreliable.

## Results

A total of 52 individuals were equipped with TDRs of which 19 were females and 33 were males. Sampling was fairly evenly distributed over the three years of study: 16 (7 female) in 1995/6, 15 (5 female) in 2005/6 and 21 (7 female) in 2009/10. The diveMove analysis recognised a total of 429 foraging trips and 11,095 dives from these TDR records. 

The residual variance in dive depth from the global model was significantly heterogeneous between sexes (LR = 5003.2, df = 1, P < 0.0001). Removal of the trip and individual terms from the global model resulted in significant increases in variance (trip: LR = 3571.7, df = 1, P < 0.0001; individual: LR = 208.9, df = 1, P < 0.0001) and so trip nested within individual was the most parsimonious random effects structure. Removal of the mass x sex interaction term produced no significant increase in variance (mass x sex: LR = 0.8, df = 1, P > 0.3; mass: LR = 0.9, df = 1, P > 0.3), indicating that the slope of the mass-depth regression was similar for the two sexes, and so this effect was removed. Removal of the sex term from the additive sex + mass model did not produce a significant increase in variance (LR = 0.58, df = 1, P > 0.4), whereas removal of the mass term produced a marginally significant increase (LR = 4.2, df = 1, P < 0.05). However, models containing either mass or gender separately both explained a highly significant amount of the variance compared to the model with only random effects (mass: LR = 21.7, df = 1, P < 0.0001; sex: LR = 18.0, df = 1,P < 0.0001). Mass differed highly significantly between the two sexes (LR = 17236.0, df = 1, P < 0.0001) with females being 0.5 kg lighter on average than males, so the effects of these two variables on dive depths were confounded.

**Figure 1:**
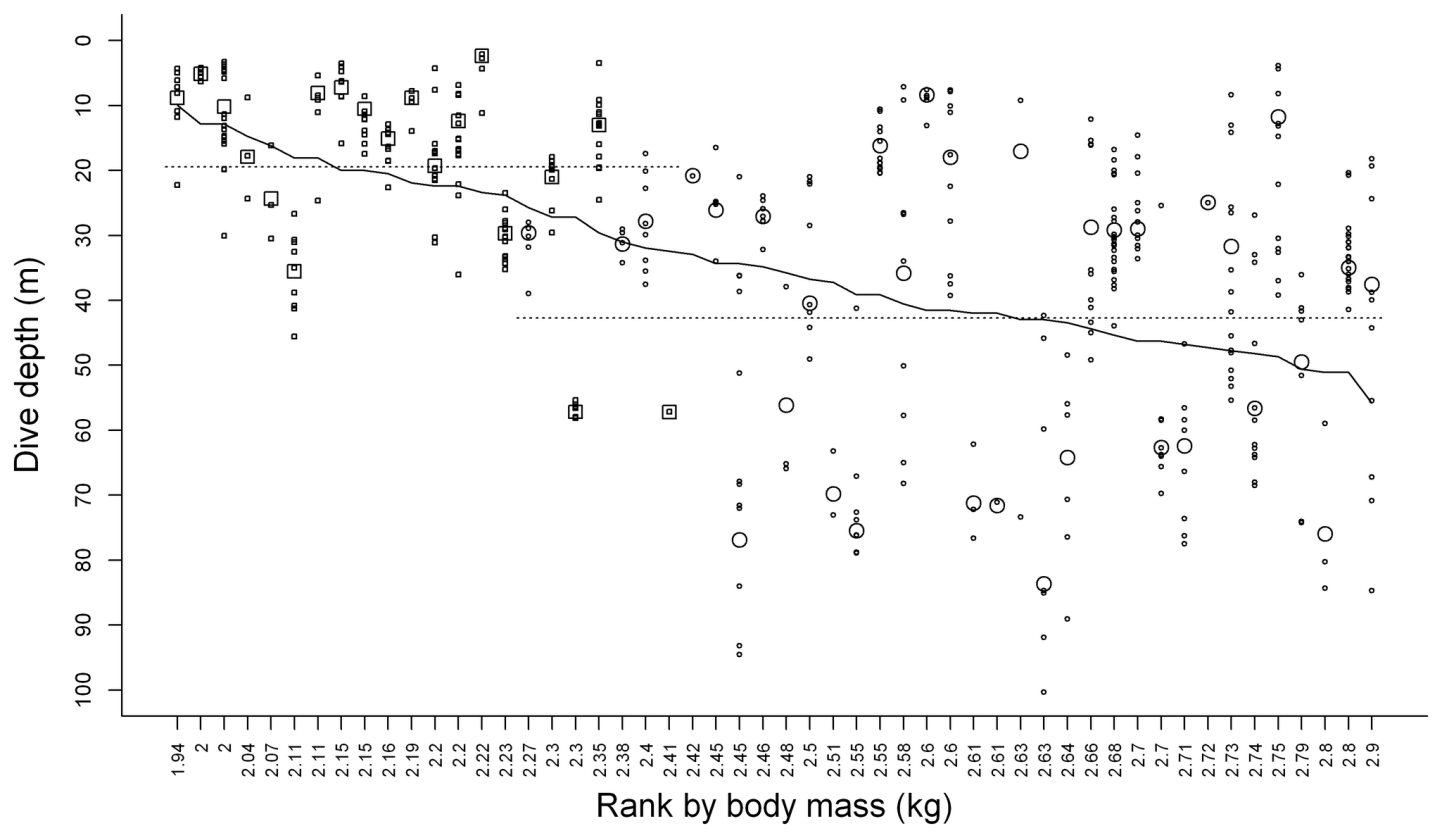
Dive depths of South Georgia Shags in relation to sex, ranked body mass and trip-within-individual variation. Squares represent females and circles males. The large symbols indicate the average dive depth by an individual and the small symbols the average by each trip it made, taken from the fitted values of the selected model. Dotted lines indicate the average dive depths for females (19.4 m) and males (42.7 m). The solid line represents the best-fit regression of dive depth against mass from the model without sex included (equation of the line, depth = -82.7 + 47.7 x mass (kg)), note that the plotted slope is irregular as mass is on a ranked scale to prevent overlap of points for individuals such that mass increments across tick intervals are variable.

The selected models therefore included either sex or mass, trip nested within individual and a sex-specific residual variance structure. Average dive depths of females was 19.4 m ± 5.07 and that of males was twice as deep at 42.7 m ± 3.1, while dive depths increased with mass at a rate of 47.7 m kg^-1^ ± 9.3. Examination of the fitted values from the selected models (Figure 1) illustrates the separate effects of sex and mass upon dive depths. This also reveals substantial departures in average individual dive depths from the values predicted by the fixed sex or mass parameters, and that males tend to exhibit greater between and within individual variability than females. 

Variance components analysis revealed that, for the random effects only model, 45.5% of the variance occurred among individuals, 22.6% among trips and 31.8% among dives. The R^2^ approximation showed that the fixed sex effect explained 31.4% of the variation among individuals and mass 55.9%. When mass was added to the model already containing the fixed sex effect, the R^2^ approximation showed that mass explained 5.8% of the individual variation. However, as there is evidence for variance differing between sexes at all levels of the sampling hierarchy we fitted random effects models of trip within individual for each sex separately and calculated variance components for each [45]. Males exhibited greater variability in their dive depths compared to females: male standard deviations were 1.18 times higher at the individual level, 3.26 times higher for trips within individuals and 2.80 times higher for dives within trips, while individual repeatability for females was almost double that of males (Table 1). R^2^ approximations within sexes showed that the fixed effect of mass explained 0.8% of the individual level variance in males and 24.1% in females.

**Table 1:** Variance components of South Georgia shag dive depths of based on the trip nested within individual random effects model, calculated for each sex separately.

Variance component	Male (n = 33)			Female (n = 19)		
	σ^2^	σ	σ^2^ %	σ^2^	σ	σ^2^ %
Individual	313.8	17.7	41.5	221.6	14.9	82.0
Trip	246.3	15.7	32.6	23.1	4.8	8.5
Dive	196.5	14.0	26.0	25.5	5.0	9.4

σ^2^ % represents the proportion of the total variance within each sex that is explained by the given variance component: the value for the individual level gives an estimate of individual repeatability or specialisation.

## Discussion

Sex differences in dive depths have been found in a wide range of air-breathing, diving vertebrate taxa, including spheniscids [46,47], phalacrocoracids [48], sulids [9,49,50], alcids [51,52], otarids [53] and phocids [17,54]. We found that male South Georgia shags on average dived deeper than females, which agrees with numerous studies of other members of the blue-eyed shag complex [26,27,29,31]. This pattern is not universal however: female Kerguelen shags *P. verrucosus* dive almost as deep as males around colony sites where little shallow water is available [32] and a study of Antarctic shag *P. bransfieldensis* found females dived deeper than males [55].

Individual repeatability in the dive depths of air-breathing vertebrates has been found in sphensicids [56], phalacrocoracids [31,57-59], alcids [52,60], mustelids [61], otarids [18,62], phocids [17] and odontocetes [63]. In our study, we found an exceptionally high level of individual repeatability in female shags, which was almost double that of males. Individual females exhibited greater fidelity to depths both on successive foraging trips and dives within trips than males, which were more flexible in their depth selection. These different patterns of individual variation within sexes are consistent with those found for Macquarie shags *P. purpurascens* [31] and Crozet Shags *P. melanogenis* [57]. It therefore appears that vertical niche partitioning in blue-eyed shags arises not only from gender partitioning in mean dive depths, but also from differential degrees of individual specialisation within sexes.

Cormorant dive depths increase with body size both among and within species [31,64] which have potential to reflect optimal dive depths associated with mass-specific differences in oxygen budgets, energy expenditure, movement rates and buoyancy [65-68]. Our study found that the average difference in body mass between sexes and the range in masses within sexes were both around 0.5kg, so it had the potential to explain both sex-specific and individual variation in their dive depths. We found support for this hypothesis, as the mean dive depth of South Georgia shags increased with body mass at a rate of 47.7 m kg^-1^ (almost identical to the rate of 46.5 m kg^-1^ found for Kerguelen shags [32]), which accounted for all of the sex-specific variation and over half of the individual variation. However, owing to confounding of the body mass and sex effects we cannot discount the possibility that sex-specific foraging strategies might be responsible instead. When controlling for sex, the depth versus body mass regression slope was only marginally significant and similar for males and females. For males, the individual variation around this regression line was large and mass only explained 0.8% of the variation, suggesting that individual foraging strategies rather than allometry was the main determinant of individual variation in dive depth. In contrast, female individual dive depths were more tightly clustered around the regression line and mass explained almost a quarter of the variation, suggesting that allometry may play a more important role in influencing their dive depths. The physiological or mechanical processes that give rise to these inconsistent allometric patterns among sexes warrant further investigation. 

We found no annual variation in sex-dependant dive depths across the three widely spaced years of our study. This contrasts with studies of other shag species elsewhere in which males switched from deep benthic diving to shallower pelagic diving during years of high epipelagic fish abundance with a consequent reduction in the sex-specific differences in dive depths [58,69,70]. The lack of annual variability in dive depths in our study is likely to result from an absence of epipelagic fish in the Southern Ocean [71] such that shags there have no alternative but to forage benthically. No studies have followed the diving behaviour of individual cormorants across years, so the degree of retention of individual foraging specialisation across years is unknown. However, a study of Brünnich’s guillemots *Uria lomvia* found that individuals did maintain specialisation in dive depths across years, although the consistency in behaviour was greater within years, suggesting that birds adopted long-term foraging strategies but modified them in response to annual variations in prey distribution [52].

Sex and individual niches in cormorants may be partitioned along other dimensions of the niche hypervolume and these may interact with depth niche partitioning. Dive depths may be related to foraging patch choice by sexes and individuals, particularly in benthic foragers. Sex-specific differences in dive depths of cormorants are associated with males foraging in deeper offshore waters compared to females [29,68,69]. Individual specialisation in dive depths can arise where a number of patches of differing water depth are available around a colony and individual cormorants have a tendency to forage repeatedly at a subset of these [30,58,72]. However, such patterns may arise via other mechanisms since individual pelagic cormorants *P. pelagicus* specialise in either shallow pelagic or deep benthic dives whilst feeding over the same patch [59]. Variation in dive depths among sexes may also be related to diet composition in blue-eyed shags, with some studies finding increases in prey size with dive depth [26,28,32,73,74] and others the opposite pattern [40,55]. The diel timing of foraging trips also differs among male and female blue-eyed shags [7,8], which might in turn influence dive depth owing to its correlation with light levels [34]. Further research into these complex interactions is required to attain a complete understanding of niche partitioning by blue-eyed shags.
